# The Needs and Experiences of People With Early-Stage Dementia Using an Application for Cognitive and Physical Activation in Germany: Qualitative Study

**DOI:** 10.2196/62689

**Published:** 2024-12-10

**Authors:** Melina Klein, Alexa von Bosse, Christophe Kunze

**Affiliations:** 1 Furtwangen University, Furtwangen, Germany; 2 Care & Technology Lab, Furtwangen University, Robert-Gerwig-Platz 1, Furtwangen, Germany

**Keywords:** touch-based digital technology, gerontology, geriatric, older, elderly, aging, aged, tablet-based technology, tablet, digital care application, mHealth, mobile health, app, health app, home care setting, caring relatives, dementia, MCI, Alzheimer, mild cognitive impairment, cognition, prototype, digital health, telehealth, dementia, memory loss, patient care, patient health, patient support

## Abstract

**Background:**

The demand for support among people with dementia is increasing, while caregiving capacity is declining. As the trend of aging at home continues, technologies can help maintain the autonomy of people with dementia, enabling them to live independently for as long as possible. Furthermore, digital applications can have numerous positive biopsychosocial effects on the health of people with dementia, enhancing their physical, cognitive, and social functioning.

**Objective:**

This study aims to investigate the needs and experiences of people with dementia regarding a prototype tablet-based application designed to promote cognitive and physical activity.

**Methods:**

We conducted a methodical triangulation by combining semistructured interviews with people with dementia and external overt participant observation while testing a tablet-based application. A qualitative content analysis, as outlined by Kuckartz, was used to analyze the data.

**Results:**

Participants demonstrated varying levels of ability and prior experience with technology. While most were initially hesitant to use the tablet independently, they were more willing to try it after receiving encouragement. Some individuals required more assistance than others, indicating the need for individualized adjustments. Personal relevance to the content appeared to be crucial for cognitive tasks, as it helped to minimize overload for people with dementia. The participants appreciated social interaction with researchers and direct communication. Therefore, it is important to consider the role of personal support when developing and implementing technology.

**Conclusions:**

The successful implementation and use of technology requires acceptance and an effective interaction between people with dementia, technology, and caregivers or caring relatives providing personal support. The acceptance of the application was found to be less influenced by the types and presentation of tasks and more by content relevance and social interaction. Ideally, one-on-one support will be provided during use, though this requires additional time and financial resources, which are often limited in caregiving settings.

## Introduction

Dementia is a leading cause of disability and care dependency among older adults worldwide [1]. Currently, around 50 million people are living with dementia; projections indicate that dementia prevalence will increase to 152 million by 2050 [2]. Consequently, the demand for support for people with dementia is increasing. This may become a key challenge due to the simultaneous decrease in caregiving capacity. In Germany, most people with dementia live in their own homes, where they receive care from family members acting as caring relatives (CRs) and from caregivers [3]. These support systems play a crucial role in dementia care, often by acquiring the skills to manage challenging behaviors associated with dementia [4].

Given the current trend toward aging at home, technology can help people with dementia preserve their autonomy, allowing them to live independently in familiar environments for as long as possible [5]. Integrating technology into dementia care can assist in preserving physical functions [6] and strengthening cognitive functions [7]. Personalized digital technologies have the potential to enhance the well-being of people with dementia by improving behavior, mood, sense of identity, and social interactions [8]. A holistic biopsychosocial approach is essential to address the complex needs of people with dementia, especially considering the frequent occurrence of multimorbidity in this population, which presents additional challenges for care and support [9].

The aim of this study is to investigate the needs and requirements of people with dementia regarding the use of interactive videos on a tablet computer, including cognitive and physical tasks to maintain their independence.

## Methods

### Ethical Considerations

The study was approved by the Ethics Committee of the German Society for Nursing Science (EK-22-038), and it complies with the Declaration of Helsinki. Prior to enrollment in the study, all participants provided written informed consent. All participants were anonymized. They did not receive any compensation.

### Study Design

**Figure 1.**
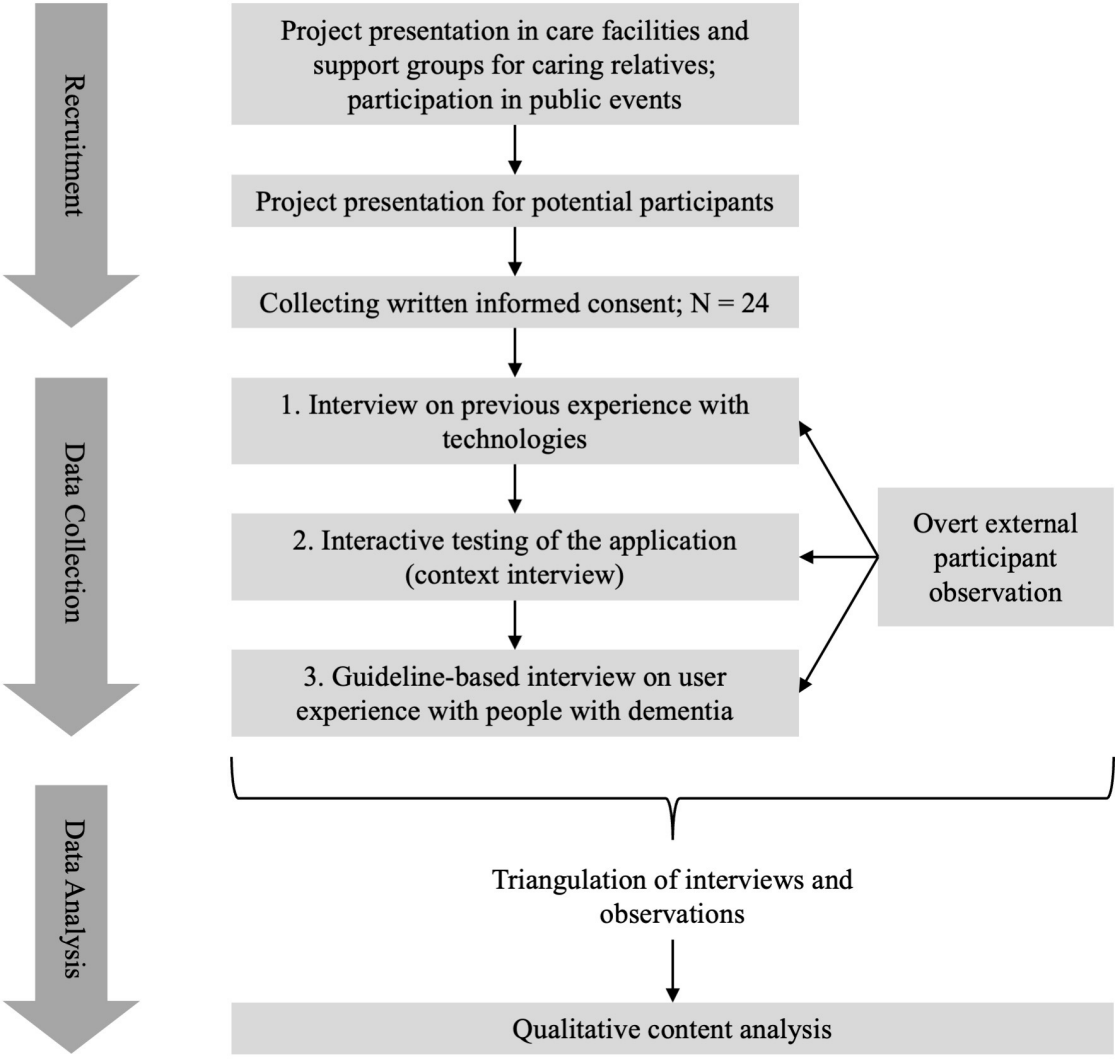
Methodical approach of presented study [11].

This study used a qualitative triangulation approach combining observations and interviews, with data from each method systematically aligned. The study was conducted in Germany and follows the SRQR (Standards for Reporting Qualitative Research) [10]. A summary of the study design and methodical approach is presented in Figure 1.

### Sampling, Recruitment, and Field Access

The study included participants aged 50-90 years with mild cognitive impairment or early-stage dementia. They had to reside in Germany and were required to have sufficient verbal communication skills and adequate vision and hearing. Previous experience with mobile devices was intentionally not set as an inclusion criterion. Recruitment and access to participants were achieved by attending events; presentations in support groups; and visits to outpatient, semi-inpatient, and inpatient care facilities in Southwest Germany.

### Data Collection

Data collection was done in day care centers or in participants’ homes, using overt, external participant observations and guided context-driven interviews, which were recorded using a sound recorder. The process of data collection was divided into three phases:

1. A discussion of the participant’s previous experience with digital technologies
2. Interactive testing of the application on a tablet computer (see Multimedia Appendix 1 for a section of the application’s content)
3. A guideline-based interview to assess the user experience

We selected the interview questions considering the limited cognitive abilities of the participants. Therefore, they were formulated in a language that was easy to understand. Textbox 1 provides a summary of the key interview topics, along with representative examples of the types of questions posed.

Textbox 1. **Key interview topics and representative questions**

1. Prior experience and personal characteristics (age, ownership of a smartphone or tablet, use of a smartphone or tablet in daily life, reasons for utilisation or non-utilisation)

 eg, Have you ever used a tablet computer before?

2. Overall evaluation of usage of the application (acceptance-rejection)

 eg, How did you like using the tablet?

3. Usability and media presentation of the application (operability, design and layout, language)

 eg, How clear was the sound?

4. Content of the application (evaluation of interaction content, evaluation of task types, personal content preferences)

  eg, Which exercise did you like the most?

Each main question was followed by additional questions to gain a deeper insight into the context and motivations behind the participants’ responses, ensuring a comprehensive understanding of their perspectives. We used a variety of open-ended and closed questions.

Two researchers conducted the data collection process. One researcher provided support during the use of the application by offering verbal assistance and aiding in the execution of the tasks to promote its use (Multimedia Appendix 2). The second researcher observed the process and made field notes based on predefined criteria, including attention span, facial expressions, and gestures, during the use of the application.

### Data Analysis

After transcribing the meaning of the collected data, we carried out a qualitative content analysis according to Kuckartz [11]. The observation protocols were triangulated with the interviews. This allowed for unspoken aspects to be included in the analysis. Following the initial coding, related codes were grouped into categories, facilitating data organization and pattern recognition. Broader themes emerged through categorization, which was essential for understanding the data’s underlying meaning. During data analysis, we supplemented deductive categories with inductive categories (Figure 1).

## Results

### Participants’ General Perceptions of the Technology and Their Usage Patterns

The characteristics of the participants are presented in Table 1. The participants showed great heterogeneity in terms of skills and previous experience with technology, resulting in varying levels of proficiency in using a tablet computer. This is likely influenced by their prior experience, such as owning or not owning a mobile device. Overall, the participants demonstrated a high level of engagement while using the application, as indicated by focused attention on videos and comments related to the content. A majority of the participants expressed positive feedback regarding the application; however, many were unable to envision using the tablet computer independently.

**Table 1.** Profiles of people with dementia.

Study variables	Participants (N=24), n (%)
Sex	
Male	10 (42)
Female	14 (58)
Owning a smartphone or tablet	
Yes	10 (42)
No	14 (58)
Using a smartphone or tablet	
Often	5 (21)
Rarely	5 (21)
Never	14 (58)

### Role of Technology Use

Most participants were proficient in recognizing visual elements, including both images and videos. However, observations regarding the varying effects of auditory stimuli on participants highlighted the challenge of designing an application that is balanced and accessible to different user groups.

### Role of the Content Within the App

#### Cognitive Tasks

When evaluating preferences for task types, the participants demonstrated indifference toward task types, including arithmetic, pictorial, and auditory tasks. Instead, they were more engaged when the content was personally relevant to their experiences. Tasks within the application fell into three categories: those that can be solved based on personal experience, those that can only be solved in the context of the story, and those that require acquired knowledge or skills.

#### Personal Experiences

The individual experiences of people with dementia played a crucial role in recognizing locations depicted in the application scenes. In particular, visual and haptic experiences, such as walking through a meadow and associated memories of the perceived feeling, acted as triggers for verbal expressions from the participants. Familiar memories evoked by the content may have fostered interest and concentration.

### Role of Personal Support During Use

Initially, most participants were hesitant to use the tablet independently but were willing to try it after receiving positive reinforcement from the researcher.

It was noticeable that the participants often sought contact with the researchers for personal support and reassurance, frequently sharing personal stories, even while watching videos or receiving task instructions. This highlighted a strong desire for communication and social interaction while using the application.

**Figure 2.**
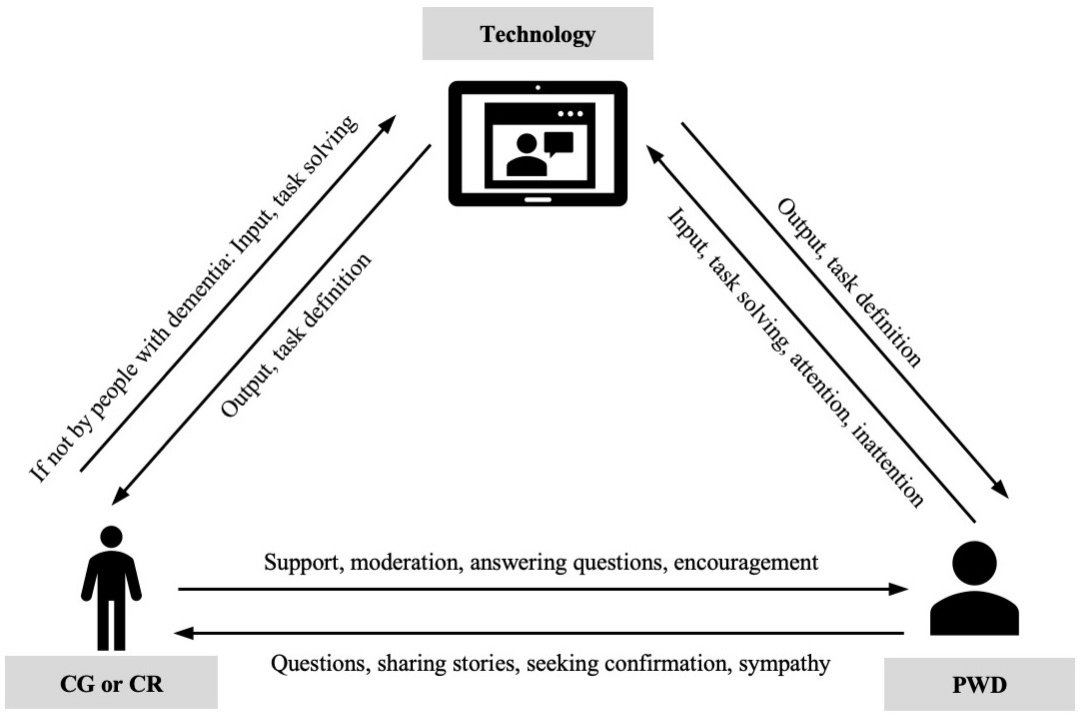
Interaction between technology, PWD, and CGs or CRs [12]. CG: caregiver; CR: caring relative; PWD: people with dementia.

When uncertain, the participants appreciated clear instructions from the researchers and were not hesitant to ask further questions (Figure 2). Data suggests that these positive feelings toward the researchers—whether, derived from sympathy, their presence or social interaction likely influenced their perceived acceptance and evaluation of the application positively. The participants’ politeness also contributed to their willingness to engage with the application and give positive feedback. In addition, participation in the study and the resulting change in the participants’ daily routine may have positively influenced the overall evaluation of the application.

## Discussion

### Principal Results

The findings indicate that personal support is crucial for a successful overall interaction while using the application. If it is not possible for people with dementia to use the application independently, alternative scenarios for use must be considered. One such scenario is one-to-one support by CRs or caregivers, which fosters social interaction. This support can motivate users and encourage device use, highlighting the relational aspects of care that are important for people with dementia. However, providing direct support requires time, a resource that is often limited for caregivers [13]. Additionally, one-to-one support can be a financial burden, as CRs perceive the associated expenses as high and, consequently, may refrain from using the application [14].

We discovered that the type of task and the visual presentation of cognitive tasks (image-based, text-based, or auditory) were secondary in importance. Instead, the content of the tasks played a more crucial role. To develop tasks that activate cognition, it is necessary to design content that establishes an emotional connection for people with dementia with their prior experiences and interests. This approach is fundamental for the acceptance of the technology. Additionally, including relational aspects, such as sharing relevant anecdotes or engaging in social interaction, further enhances the effectiveness of the technology and its acceptance by people with dementia.

### Limitations

The reliability of the study may be reduced due to participants potentially adopting a desirable response and behavior during interviews. To address this, we triangulated the data by including observations along with mutual verification among the researchers. The researchers’ reflexivity regarding their own roles in the study ensures a higher level of objectivity.

### Comparison With Prior Work

In a previous study, expert interviews were conducted to analyze the requirements for an application for people with dementia. The application tested in the current study was designed based on those findings [15].

According to our study, acceptance is the base for a successful engagement with the application. A systematic review reported similar results, emphasizing the importance of acceptance [6]. For a successful engagement, social context is another crucial factor, as highlighted by our study findings. Smith et al [16] discovered that technology use is perceived more as a social event by people with dementia when they are in the presence of others or in groups. The tablet computer serves as a catalyst for conversation, facilitating the exchange of interesting anecdotes [16].

Woods et al [17] demonstrated that biographical reference plays an important role in solving cognitive tasks for people with dementia. Although they often struggle to recall recent events, they are able to retain childhood memories. Tasks that include biographical references can be easier for people with dementia, as they include leveraging their cognitive strengths and minimizing overload.

### Conclusions

While designing an application for patients with dementia, it is important to consider the heterogeneity in this group and dependence on their daily fluctuating cognitive state. Therefore, providing opportunities for individualized adaptation of the technology is crucial for addressing diverse interests and abilities. Biographical content-related tasks can have a positive impact on cognitive activation. It is therefore reasonable to suggest that such interventions linked to biography could also be employed in individuals with other neurodegenerative diseases with the objective of preserving cognitive abilities and memories.

The participants in this study expressed a common interest in social interaction. Therefore, it is important to consider the significant role of personal support when developing and implementing technology, and ensuring easy access. Potential users, CRs, and caregivers should face as few barriers as possible when learning, purchasing, and using the application. This is particularly important, given the short period of use due to the changing cognitive state. If these conditions are met, this application has the potential to promote independent living among people with dementia.

## Supplementary material

10.2196/62689 Multimedia Appendix 1 A section of the application’s content.

10.2196/62689 Multimedia Appendix 2 The personal support that was provided while using the application.

## Abbreviations

CR: caring relative
SRQR: Standards for Reporting Qualitative Research
