# Supplementary figures and images for: The Needs and Experiences of People With Early-Stage Dementia Using an Application for Cognitive and Physical Activation in Germany: Qualitative Study
Source: JMIR Aging. 2024 Dec 10;7:e62689. doi: 10.2196/62689 (PMC11651421; doi:10.2196/62689)

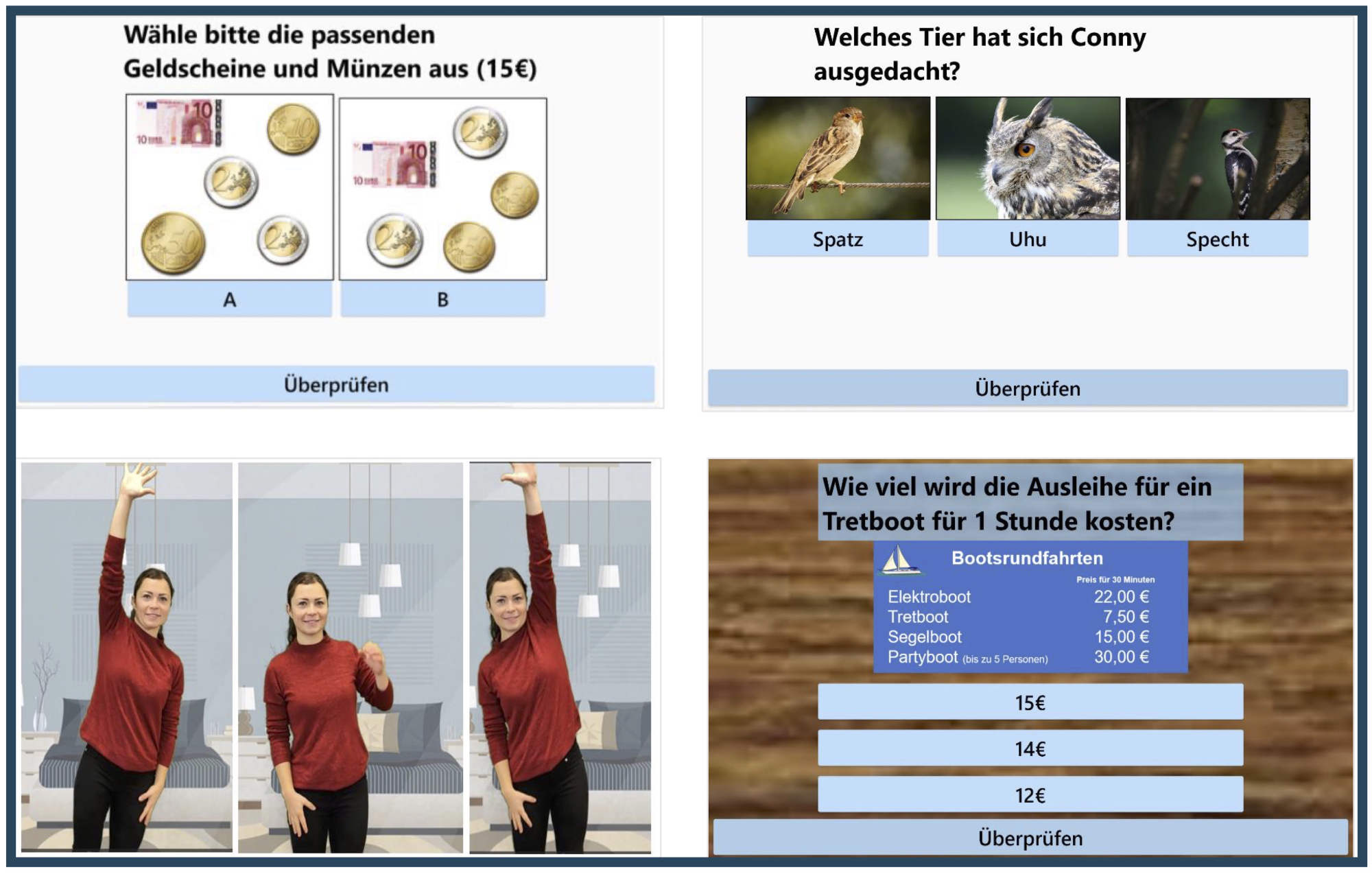

Supplement: Multimedia Appendix 1 [file aging-v7-e62689-s001.png]

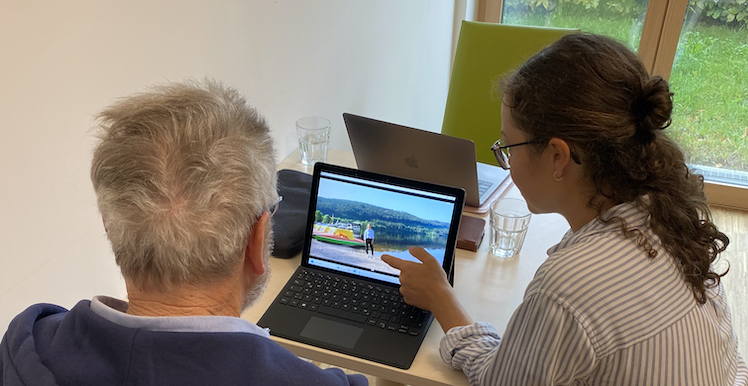

Supplement: Multimedia Appendix 2 [file aging-v7-e62689-s002.png]
